# Draft Genome Sequence of the Yeast *Pichia manshurica* YM63, a Participant in Secondary Fermentation of Ishizuchi-Kurocha, a Japanese Fermented Tea

**DOI:** 10.1128/MRA.00528-19

**Published:** 2019-07-03

**Authors:** Takahito Toyotome, Miyu Yamamoto, Masanori Horie

**Affiliations:** a Department of Veterinary Medicine, Obihiro University of Agriculture and Veterinary Medicine, Obihiro, Hokkaido, Japan; b Diagnostic Center for Animal Health and Food Safety, Obihiro University of Agriculture and Veterinary Medicine, Obihiro, Hokkaido, Japan; c Medical Mycology Research Center, Chiba University, Chiba, Japan; d Health Research Institute, National Institute of Advanced Industrial Science and Technology (AIST), Takamatsu, Kagawa, Japan; Vanderbilt University

## Abstract

Pichia manshurica is common in fermentation; however, genome analysis has never been reported for the species. This yeast plays a role in the secondary fermentation of Ishizuchi-kurocha, a traditional Japanese fermented tea. This paper presents the draft genome sequence of P. manshurica YM63, isolated from the leaves of fermented tea.

## ANNOUNCEMENT

Pichia manshurica (phylum Ascomycota, subphylum Saccharomycotina) is a common yeast found in fermented animal feed and foods such as fermented maguey juice (1), a traditional beverage of Burkina Faso (2), wines (3), and silages (4). Ishizuchi-kurocha is a traditional fermented tea in Shikoku Island, Japan (5). The yeast species *Pichia manshurica* naturally occurs during the production process of Ishizuchi-kurocha. The yeast species has important roles in fermentation and rotting plant materials. The genome sequences of Pichia kudriavzevii (6, 7) and Pichia membranifaciens (8), which are closely related to P. manshurica, and their comparative analysis (9) have been presented. Genome analysis of *P. manshurica*, however, has not been reported.

We used strain YM63, isolated from leaves in the secondary fermentation step of Ishizuchi-kurocha production. The strain was confirmed to be the correct species by 28S rRNA sequencing. YM63 was cultured in yeast extract-peptone-dextrose broth at 30°C for 14 h with shaking at 130 rpm. We prepared genomic DNA from the YM63 strain using a blood and cell culture DNA minikit (Qiagen, Inc.). Genome sequencing was performed using a MiSeq platform (Illumina, Inc.) and GridION with flow cell type R9.4.1 (Oxford Nanopore Technologies). For MiSeq sequencing, genomic DNA was sheared with a Covaris S2 sonicator (Covaris, Inc.) to obtain ∼500-bp DNA fragments. A library was prepared from 200 ng of fragmented DNA using a preparation kit (Kapa HyperPrep kit; Kapa Biosystems) and an adapter kit (FastGene adapter kit; Nippon Genetics Co. Ltd.). After quantification and qualification, the prepared library was sequenced with MiSeq technology to produce 2 × 151-bp paired-end reads. A total of 2,327,748 reads with a Q30 of 83.4% were obtained by MiSeq sequencing. For GridION analysis, a library was prepared using a ligation sequence kit (Oxford Nanopore Technologies). By GridION analysis, 749,822 reads (average length, 16,203 bp) were obtained. MiSeq reads were trimmed using the parameters -q 20 -l 127 with Sickle v1.33 (10). A total of 2,170,013 filtered reads were used for a subsequent assembly. Adaptor sequences in the reads from GridION were trimmed with Porechop v0.2.3, and the trimmed reads were quality filtered using the parameters –min_mean_q 80.05 –min_length 1000 with Filtlong v0.2.0. Error-prone read data from GridION were processed using Canu v1.8 (11). A total of 668,602 reads was used for the subsequent assembly. Finally, assembly was done using MaSuRCA v3.2.8 (12), and polishing was done using Bowtie 2 v2.3.4.1 (13) and Pilon v1.22 (14).

The draft genome includes eight contigs with a total size of 12,405,322 bp. The GC content is 42.3%. The *N*_50_ contig and the longest contig sizes are 2,862,201 bp and 3,465,259 bp, respectively. The total contig size of *P. membranifaciens* KS47-1 is 11.4 Mb (8). The genome assembly size of *P. membranifaciens* NRRL Y-2026 is 11.5 Mb (9), which is slightly smaller than that of *P. manshurica* YM63. According to BLAST homology analysis, the scf7180000000032 contig (GenBank accession no. BJFO01000006) is estimated to be a mitochondrial genome. Benchmarking Universal Single-Copy Orthologs (BUSCO) v3 (15, 16) analyses using Ascomycota and Saccharomycetales data sets showed 83% and 80.5% complete BUSCO, respectively.

### Data availability.

The draft genome sequence of *Pichia manshurica* YM63 was deposited in DDBJ/ENA/GenBank under accession no. BJFO01000001 to BJFO01000008. The raw sequencing reads were submitted to the DRA under accession no. DRA008229.

## References

[1] Nolasco-Cancino H, Santiago-Urbina JA, Wacher C, Ruíz-Terán F 2018 Predominant yeasts during artisanal mezcal fermentation and their capacity to ferment maguey juice. Front Microbiol 9:2900. doi:10.3389/fmicb.2018.02900. 30574125 PMC6291486

[2] Sanata B, Adama Z, Ibrahim S, Apoline S, Mamoudou C, Constant S, Robert GT, Christophe H 2017 Characterization of the fungal flora of dolo, a traditional fermented beverage of Burkina Faso, using MALDI-TOF mass spectrometry. World J Microbiol Biotechnol 33:172. doi:10.1007/s11274-017-2335-1. 28852966

[3] Ramírez-Castrillón M, Mendes SDC, Valente P 2017 South Brazilian wines: culturable yeasts associated to bottled wines produced in Rio Grande do Sul and Santa Catarina. World J Microbiol Biotechnol 33:77. doi:10.1007/s11274-017-2244-3. 28341906

[4] Wang H, Hao W, Ning T, Zheng M, Xu C 2018 Characterization of culturable yeast species associating with whole crop corn and total mixed ration silage. Asian-Australas J Anim Sci 31:198–207. doi:10.5713/ajas.17.0183. 28728388 PMC5767501

[5] Horie M, Nara K, Sugino S, Umeno A, Yoshida Y 2017 Comparison of antioxidant activities among four kinds of Japanese traditional fermented tea. Food Sci Nutr 5:639–645. doi:10.1002/fsn3.442. 28572952 PMC5448361

[6] Chan GF, Gan HM, Ling HL, Rashid N 2012 Genome sequence of *Pichia kudriavzevii* M12, a potential producer of bioethanol and phytase. Eukaryot Cell 11:1300–1301. doi:10.1128/EC.00229-12. 23027839 PMC3485917

[7] Douglass AP, Offei B, Braun-Galleani S, Coughlan AY, Martos AAR, Ortiz-Merino RA, Byrne KP, Wolfe KH 2018 Population genomics shows no distinction between pathogenic *Candida krusei* and environmental *Pichia kudriavzevii*: one species, four names. PLoS Pathog 14:e1007138. doi:10.1371/journal.ppat.1007138. 30024981 PMC6053246

[8] Konishi M, Arakawa T, Kato Y, Ishida M, Horiuchi J 2017 Draft genome sequencing of ascomycetes yeast *Pichia membranifaciens* KS47-1, which shows high acetate resistance in lignocellulosic feedstock hydrolysate. Genome Announc 5:e01672-16. doi:10.1128/genomeA.01672-16. 28232447 PMC5323626

[9] Riley R, Haridas S, Wolfe KH, Lopes MR, Hittinger CT, Göker M, Salamov AA, Wisecaver JH, Long TM, Calvey CH, Aerts AL, Barry KW, Choi C, Clum A, Coughlan AY, Deshpande S, Douglass AP, Hanson SJ, Klenk H-P, LaButti KM, Lapidus A, Lindquist EA, Lipzen AM, Meier-Kolthoff JP, Ohm RA, Otillar RP, Pangilinan JL, Peng Y, Rokas A, Rosa CA, Scheuner C, Sibirny AA, Slot JC, Stielow JB, Sun H, Kurtzman CP, Blackwell M, Grigoriev IV, Jeffries TW 2016 Comparative genomics of biotechnologically important yeasts. Proc Natl Acad Sci U S A 113:9882–9887. doi:10.1073/pnas.1603941113. 27535936 PMC5024638

[10] Joshi N, Fass J 2011 Sickle: a sliding-window, adaptive, quality-based trimming tool for FastQ files (version 1.33).

[11] Koren S, Walenz BP, Berlin K, Miller JR, Bergman NH, Phillippy AM 2017 Canu: scalable and accurate long-read assembly via adaptive k-mer weighting and repeat separation. Genome Res 27:722–736. doi:10.1101/gr.215087.116. 28298431 PMC5411767

[12] Zimin AV, Marçais G, Puiu D, Roberts M, Salzberg SL, Yorke JA 2013 The MaSuRCA genome assembler. Bioinformatics 29:2669–2677. doi:10.1093/bioinformatics/btt476. 23990416 PMC3799473

[13] Langmead B, Salzberg SL 2012 Fast gapped-read alignment with Bowtie 2. Nat Methods 9:357–359. doi:10.1038/nmeth.1923. 22388286 PMC3322381

[14] Walker BJ, Abeel T, Shea T, Priest M, Abouelliel A, Sakthikumar S, Cuomo CA, Zeng Q, Wortman J, Young SK, Earl AM 2014 Pilon: an integrated tool for comprehensive microbial variant detection and genome assembly improvement. PLoS One 9:e112963. doi:10.1371/journal.pone.0112963. 25409509 PMC4237348

[15] Waterhouse RM, Seppey M, Simao FA, Manni M, Ioannidis P, Klioutchnikov G, Kriventseva EV, Zdobnov EM 2018 BUSCO applications from quality assessments to gene prediction and phylogenomics. Mol Biol Evol 35:543–548. doi:10.1093/molbev/msx319. PMC5850278 29220515

[16] Simão FA, Waterhouse RM, Ioannidis P, Kriventseva EV, Zdobnov EM 2015 BUSCO: assessing genome assembly and annotation completeness with single-copy orthologs. Bioinformatics 31:3210–3212. doi:10.1093/bioinformatics/btv351. 26059717

